# Pretransplant Cardiac Evaluation Using Novel Technology

**DOI:** 10.3390/jcm8050690

**Published:** 2019-05-16

**Authors:** Mohamad Hemu, Allison Zimmerman, Dinesh Kalra, Tochukwu Okwuosa

**Affiliations:** Department of Medicine, Rush University Medical Center, Chicago, IL 60612, USA; mohamad_r_hemu@rush.edu (M.H.); Allison_C_Zimmerman@rush.edu (A.Z.); Dinesh_Kalra@rush.edu (D.K.)

**Keywords:** hematopoietic stem cell transplant, cardiac imaging, cardiovascular complications, echocardiogram, cardiac magnetic resonance, speckle tracking echocardiography, coronary computed tomography

## Abstract

Hematopoietic stem-cell transplantation (HSCT) is a complex procedure that has been increasingly successful in treating malignant and nonmalignant conditions. Despite its effectiveness, it can be associated with potentially life-threatening adverse effects. New onset heart failure, ischemic disease, and arrhythmias are among the most notable cardiovascular complications post-HSCT. As a result, appropriate cardiac risk stratification prior to transplant could result in decreased morbidity and mortality by identifying patients with a higher probability of tolerating possible toxicities associated with HSCT. In this review, we aim to discuss the utility of cardiac screening using novel modalities of imaging technology in the pre-HSCT phase.

## 1. Introduction

The most notable complications related to high-dose chemotherapy or irradiation in the conditioning phase are cardiovascular in nature [1]. These adverse cardiovascular outcomes can occur acutely within the first few months of treatment or many years after transplantation. However, the true incidence of cardiovascular complications immediately following hematopoietic stem cell transplant (HSCT) is not well established. A large single-center retrospective study evaluated the incidence of cardiac arrhythmias in patients who underwent HSCT. Among 1177 study participants, 104 developed arrhythmias, mainly supraventricular in nature. These patients were at an increased risk for one-year mortality post-transplant. They also had longer hospital stay and greater intensive care unit admissions [2]. Another retrospective study examined risk factors associated with development of atrial fibrillation (AF) immediately post-HSCT. In this study, 27% of patients developed AF at a mean duration of 14.8 days following HSCT. A dilated left atrium, left ventricular dysfunction, and hypertension pre-HSCT were significantly associated with the development of atrial fibrillation in the peri-HSCT period [3]. On the other hand, serious complications, such as large pericardial effusions with cardiac tamponade, remain rare, occurring in less than 1% of patients [4]. However, new onset congestive heart failure (CHF) remains a major concern in patients receiving HSCT with high dose cyclophosphamide (CY). It is estimated that 28% of patients who received high dose CY were subsequently diagnosed with CHF [5,6]. This problem can be attenuated by the introduction of low dose regimens that were shown to decrease the incidence of new onset CHF to less than 2% [7].

Cardiac complications associated with HSCT can manifest even years after transplantation [8,9]. HSCT recipients have increased risk of mortality from cardiovascular specific causes (3.6 per 1000 person-years) [10]. Additionally, the cumulative incidence of cardiovascular disease (CVD), including coronary artery disease (CAD), cerebrovascular disease, and heart failure, approaches 23% at 25 years after HSCT, and a 7.0 to 15.9-fold increased risk of CVD risk factors such as hypertension, diabetes, and dyslipidemia was observed within eight years post-transplant [9,11,12,13]. Additionally, patients who received anthracyclines have increased risk for CHF with an incidence of 4.8% at five years and up to 9.1% at 15 years [14].

Malignancies and CVD share similar risk factors [15]. As a result, HSCT patients may already have impaired cardiovascular function prior to HSCT. One study identified 2430 patients who underwent HSCT. Compared to controls, HSCT patients had increased prevalence of hypertension, diabetes, smoking, CAD, peripheral vascular disease, and heart failure at baseline [16]. Moreover, the selection criteria for HSCT are now less restrictive compared to a decade ago, and elderly patients with impaired baseline cardiac function are now considered candidates for HSCT. As a result, appropriate cardiac risk stratification prior to transplant could result in decreased morbidity and mortality by identifying patients with a higher probability of tolerating possible toxicities associated with HSCT. 

In this review, we aim to discuss the utility of cardiac screening using novel modalities of imaging technology in the pre-HSCT phase.

## 2. Current State of Cardiovascular Screening for Cardiac Structure and Function Prior to HSCT

While early life-threatening cardiac complications such as large pericardial effusion, cardiac tamponade, acute heart failure, and life threatening arrythmias following HSCT are rare [17,18,19], there is still significant risk for subacute cardiac dysfunction even in patients with apparent normal cardiac function at baseline. Optimal screening protocols for such patients have not been fully established. In general, patients with uncontrolled CHF, CAD, or arrythmias are generally not considered candidates for HSCT. The cutoff point for cardiac dysfunction depends largely on the conditioning regimen. For most institutions, a left ventricular ejection fraction (LVEF) of ≥45% is acceptable prior to using cyclophosphamide-based regimens or total body irradiation for both allogenic and autologous HSCT [20]. However, select institutions may even consider a lower LVEF cutoff in patients with limited CVD risk factors such as smoking, hyperlipidemia, CAD, arrythmia, or prior infarction [21].

Currently, the most widely used cardiac screening modality prior to HSCT is 2-dimensional echocardiography (2D echo) [22]. This cardiovascular imaging modality is often used in clinical practice as first line for screening patients for decreased LVEF. However, despite its overwhelming clinical utility, estimating LVEF using 2D echo is not without important limitations. First, it is influenced by several factors including heart rate, loading conditions, and breathing during capture of the image, all of which can lead to artifact that could alter the endocardial border. Second, it is not considered sensitive enough to detect subclinical myocardial damage, which may have major therapeutic implications. Third, 2D echo is largely operator-dependent and can result in significant interobserver variability [23,24]. As a result, clinicians who monitor changes in LVEF over time may have difficulty determining whether changes in LVEF are clinically significant or purely due to measurement error. Given these significant limitations, the use of more reliable cardiac screening modalities with limited interobserver variability and ability to detect subclinical cardiac injury is needed in HSCT patients receiving cardiotoxic agents.

## 3. Contemporary Techniques for Screening for Cardiac Structure and Function Prior to HSCT 

### 3.1. Speckle-Tracking Echocardiography

Currently, a reduction in LVEF is considered a marker of cardiotoxicity related to the chemotherapy phase of HSCT [25]. However, LVEF reduction occurs late in the disease process, and failure to recover systolic function at this stage can occur in up to 58% of patients [26]. Hence, identifying markers of early myocardial toxicity occurring in the setting of normal LVEF may lead to preventive strategies and ultimately minimize progression of underlying heart disease.

Speckle tracking echocardiography (STE) is a widely used technique to assess regional myocardial dysfunction. STE is a type of strain imaging used to describe local shortening, thickening, and lengthening of the myocardium [27]. Simply put, the interaction of the ultrasound beam with the myocardial tissue creates a speckle pattern or footprints. Each speckle can then be automatically tracked during the cardiac cycle. The changes in diastolic and systolic length of a speckle is represented as a strain value which provides information regarding myocardial deformation [27]. 

One of the most well studied strain modalities is global longitudinal strain (GLS). GLS represents shortening or lengthening of the myocardium from base to apex. A normal mean value of GLS among studies and different vendors varied between −16.7% to −23.6% [28,29]. A fall in GLS between 10% and 15% predicts subsequent cardiotoxicity [30]. One way to illustrate the advantage of using GLS in clinical practice is by providing an actual clinical scenario. A patient with normal baseline LVEF received cytotoxic chemotherapy and was noted to have a 6% drop in LVEF at six months follow-up, which by standard practice was not considered a clinically significant change. However, GLS measured at the same time decreased to −15.4% from baseline (−19.1%). At one-year follow-up, a significant drop in LVEF was noted, meeting the criteria for cardiotoxicity [31]. This example demonstrates the ability of STE to detect early evidence of cardiotoxicity not readily diagnosed by 2D echo.

Additionally, a multicenter study compared GLS and LVEF as biomarkers in predicting future cardiac dysfunction in patients receiving doxorubicin chemotherapy. Impairment in GLS at three months follow-up was a predictor of the development of cardiotoxicity at six months. Conversely, LVEF did not predict cardiotoxicity [32]. In a separate study, GLS was found to be an independent predictor of all-cause mortality in patients with systolic heart failure and was a superior prognosticator compared to all other echocardiographic parameters [33]. A recent study conducted by the EACVI-ASE-Industry Task Force to standardize deformation imaging showed that reproducibility of GLS measurements was adequate and; in many cases, superior to the reproducibility of LVEF [34].

To further illustrate the advantages of GLS, changes in LV function using STE in children who underwent HSCT in the setting of acute leukemia was assessed. Compared to controls, post-HSCT patients had similar LVEF using conventional echocardiography. However, STE parameters including GLS were significantly decreased in post-HSCT patients compared to controls. The authors of the study concluded that STE is a useful tool in detecting early myocardial dysfunction and may be considered as a routine imaging modality in this patient population [35]. 

Despite its overwhelming advantages, STE is not without important limitations. The most important limitation is inter-vendor variability [36]. Different machines and software can potentially produce different results. Therefore, until standardization in strain imaging is achieved, it is best to use the same vendor’s machine and software for serial evaluation of cardiac function. 

### 3.2. Cardiac Magnetic Resonance (CMR)

CMR is the gold standard imaging modality for detecting ventricular volumes and function [37]. CMR provides a detailed evaluation of ventricular size, thickness, wall motion, and ejection fraction. Multiple studies confirmed the superiority of CMR to 2D echo in detecting clinically relevant changes in LV function [38]. CMR has several additional strengths, including lack of ionizing radiation; and is not constrained by poor acoustic windows, which is often a significant limitation of echocardiography. In addition to cardiac size and function, CMR provides a noninvasive assessment of histopathological myocardial changes, allowing for recognition of early cardiac disease. 

Gadolinium (Gd)-containing contrast media is used in CMR to differentiate diseased vs. healthy myocardium. Both normal and diseased myocardium take up Gd. However, the rate of Gd washout is slower in the presence of underlying heart disease. For example, cellular lysis and edema occurring post myocardial infarction result in increased extracellular space and delayed washout of Gd. While LGE was originally developed to detect scared myocardium, it has also been found useful in diagnosing cardiomyopathies based on differences in LGE patterns. The role of CMR/LGE in HSCT patients receiving cardiotoxic agents such as anthracyclines is not well described in the literature. In one study, 22 patients with baseline normal cardiac function were investigated using CMR to measure LGE before, at three days, and at 28 days after treatment with anthracyclines. An increase in LGE >5 on day three was a significant predictor of decreased LVEF at 28 days of follow up [39]. In another study of 62 survivors of childhood cancer treated with anthracyclines, a decline in EF below normal for either the LV or RV was seen in 80% of patients at 7.8 years of follow up even though the majority of patients had only mild declines (10% below normal range). Yet the fibrosis by LGE was seen [40]. 

Thoracic aortic stiffness occurs as a consequence of aging and is considered a marker for increased overall mortality and adverse cardiovascular outcomes in the general population [41]. Using CMR, aortic stiffness can be accurately measured by estimating pulse wave velocity and aortic distensibility. Prior studies showed increased risk for worsening aortic stiffness in patients receiving anthracycline therapy [42]. A recent prospective study involving patients with hematologic malignancies who were treated with low to moderate dose anthracyclines had a normal LVEF on 2D echo despite an increased pulse wave velocity and LV systolic volumes on CMR as early as one month post treatment. At six months follow-up, 26% of these patients had a new drop in LVEF [43]. This study further highlights the utility of CMR in identifying subclinical cardiovascular disease in patients receiving cardiotoxic therapy. 

CMR myocardial T1 mapping is another powerful diagnostic parameter that detects subtle changes in extracellular matrix. Numerous studies emphasized the importance of focusing on changes that occur in the interstitium, including fibrosis, rather than purely on the structure and function of myocyte [44]. These changes often alter mechanical and electrical functions of the heart [45]. In one study, 37 patients who received anthracycline treatment were noted to have increased extracellular volume using T1 mapping compared with the control group [46]. It is unknown whether T1 signal changes in anthracycline treated patients signify increased risk for adverse cardiovascular outcomes; and more studies are needed. 

CMR is a sensitive imaging modality that allows detection of subclinical cardiovascular disease not detected by standard echocardiography. However, larger patient cohorts and longer follow-up periods are needed to evaluate CMR as a predictor of adverse cardiovascular outcomes in patients receiving cardiotoxic agents as part of HSCT. Additionally, its high cost and low availability at most centers preclude its use for serial monitoring of cardiotoxicity in patients with HSCT. 

### 3.3. D Echocardiography (3D Echo)

The evolution of 3D cardiac ultrasound dates to 1974 [47]. The 3D images were initially reconstructed sequentially from 2D images [48]. These methods, however, had many drawbacks including poor spatial resolution and time intensive. Only recently, with the introduction of matrix-array transducers, were rapid and real-time acquisition of 3D images made possible. Newer generation 3D transducers consist of thousands of active ultrasound elements that together allow for near real-time volumetric scanning and instantaneous high-quality images [49]. 3D echo is used for various purposes in clinical practice such as evaluation of cardiac function and anatomy, valvular heart disease, congenital abnormalities, and ventricular desynchrony. As mentioned previously, assessing LV function is one of the most common implications of echocardiography. While CMR is considered the gold standard for assessing LV function, several studies have suggested that 3D echo has a comparable accuracy and reproducibility of LV outcomes quantification [50].

As mentioned previously, measurement of LVEF using 2D echo is limited by lack of accuracy due to ventricular foreshortening and the use of mathematical models with geometrical assumptions to calculate volumes. Several studies have suggested that when compared to 2D echo, 3D echo allows for reduced analysis time, lower interobserver variability, and higher reproducibility [51,52].

Given the risk for cardiotoxicity with chemotherapy and the need for serial imaging, it is crucial to have reproducible and consistent results when images are acquired/analyzed by different observers. In a recent study, different echocardiographic techniques were compared for serial evaluation of LVEF in patients undergoing treatment with cardiotoxic agents. At one year of follow-up, 3D echo showed significantly lower temporal variability and desirable longitudinal reproducibility when compared with all other techniques [53]. 

Despite its several advantages, the role of 3D echo in HSCT patients is yet to be described in the literature.

**Figure 1:**
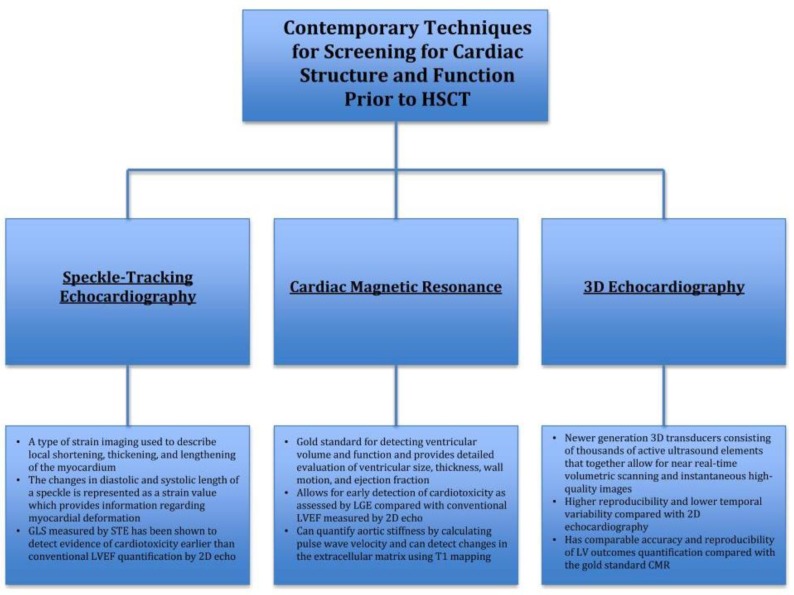
Screening for cardiac structure and function prior to hematopoietic stem cell transplant (HSCT). STE, speckle-tracking echocardiography; GLS, global longitudinal strain; LVEF, left ventricular ejection fraction; LGE, late gadolinium enhancement; LV, left ventricular; CMR, cardiac magnetic resonance; HSCT, hematopoietic stem cell transplant.

Figure 1 provides a summary of contemporary techniques for screening for cardiac structure and function prior to HSCT:

## 4. Current State of Screening for Cardiac Ischemia Prior to HSCT

### 4.1. Stress Echocardiography

Stress echocardiography involves the administration of stressors, most commonly exercise, dobutamine, and dipyrimadole, to assess the ionotropic response of the myocardium. In the cancer population, routine echocardiography is normally performed as an initial test for the assessment of CAD to depict cardiac function and rule out other diseases that may be causing the patients symptoms. However, with routine echocardiogram, regional wall motion abnormalities caused by CAD are only observed in patients with critical ischemia or prior infarction. Therefore, in patients with stable angina, stress echocardiography is acceptable, especially in those whom exercise is not feasible and those who have undergone treatment with agents associated with ischemia including 5-fluorouracil, capecitabine, bevacizumab, sorafenib, and sunitinib. Stress echocardiography has better sensitivity and specificity compared to exercise stress electrocardiography and is of great value in those patients whose stress electrocardiogram is inconclusive. Additionally, the prognostic value of revascularization therapy with interventional angiography is increased with stress echocardiogram [54]. Coronary angiography is recommended following positive stress echocardiography when wall motion defects occur at low workload, if more than five segments of the left ventricle are at risk, and if there is slow recovery or resistance to antitodes [55]. One study to date retrospectively analyzed 284 allogenic HSCT patients undergoing exercise stress echocardiography. Testing parameters included ICU admission, in-hospital death, or death within one year. Decreased resting LVEF and exercise time had a positive correlation with in-hospital death, and this was the only significant finding. Further studies are needed to determine the role of stress echocardiography in this patient population. 

### 4.2. Single-Photon Emission Computed Tomography (SPECT)

Cardiac SPECT is a noninvasive nuclear imaging modality whereby radioactive tracer, typically technetium or thallium, is intravenously injected at rest or under some form of stress (pharmacologic or exercise). The tracer is taken up by the myocardium in quantities proportional to perfusion. Therefore, in areas where perfusion is decreased (ischemia, infarction, or stress) there is a decrease in radioactive uptake. This technique is often used to evaluate for CAD, wall motion abnormalities, myocardial infarction, heart failure, and for therapy guidance [56]. It can be useful as a diagnostic tool but also has valuable prognostic utility. The advantage of nuclear imaging is the high sensitivity. Regarding cardiac complications among HSCT patients, SPECT imaging has not been studied. For cancer patients in general, nuclear imaging allows for molecular signaling to detect signs of myocardial damage including perfusion defects, cell death, and metabolic alterations [54]. Many studies that have looked at myocardial perfusion imaging in patients with cancer focus on patients that underwent radiation therapy for breast cancer or thoracic cancer. One study examined myocardial perfusion defects in patients with esophageal cancer that underwent radiation therapy and compared them to those who did not undergo radiation therapy. Fourteen of the 26 patients that underwent radiation therapy demonstrated perfusion defects on MPI. Interestingly, all 14 of these patients had distal esophageal cancer, and 11 of these 14 patients had inferior wall ischemia, suggesting a relationship between radiation therapy and the occurrence of local ischemia [57]. The drawbacks of nuclear imaging are the limited spatial resolution and exposure to radiation. However, short half-lives of thee radioactive tracers most commonly used and novel camera technology is making the dose of radiation acceptable, thus lending a possible role to nuclear imaging as a screening tool for early identification of cardiac disease in HSCT patients and cancer patients in general [58].

## 5. Contemporary Techniques for Screening for Cardiac Ischemia Prior to HSCT using Cardiac Computer Tomography (CCT)

CCT is a noninvasive approach with high sensitivity for detecting CAD and a high negative predictive value for excluding the presence of CAD. CCT enables the acquisition of thin slices (0.25 to 0.5 mm) of the heart and coronary arteries in diastole when coronary motion is minimized. Images are acquired using a multidetector (helical) CT scanner, which is more than 64 slices for coronary evaluation. Noncontrast coronary CT can also be used to quantify the coronary artery calcium score (CCS), which is a marker of atherosclerosis that is proportional to the extent of the disease. The latter has the advantage of lower radiation and lack of iodinated contrast administration but cannot visualize coronary luminal stenoses. CCT angiography (CCTA) can reliably identify more than 90% of the coronary arterial segments and in appropriately selected scenarios, can also visualize myocardial scarring based on delayed contrast enhancement and measured ejection fraction, if so desired [59]. 

## 6. Coronary Calcium Scoring (CCS)

The scanner software quantifies the amount of calcium, typically using the Agatston scoring system [60]. The current role of CCS in the general population is to refine risk in individuals who would otherwise be misclassified by standard risk assessments, such as the pooled risk cohort equation. Current guidelines suggest that CCS should be considered for asymptomatic patients with an intermediate Framingham Risk Score (FRS) for further risk stratification if they need further information regarding initiation of statin therapy [60]. Numerous studies have shown that CCS improves risk detection compared to standard risk stratification [61,62,63]. CCS testing was recently incorporated into the 2013 American College of Cardiology (ACC)/American Heart Association (AHA) risk estimator, with the suggestion to initiate statin therapy for an absolute CCS ≥300 or ≥75th percentile for age, sex, and ethnicity [64]. The NIH-NHLBI-sponsored MESA cohort evaluated the long-term ASCVD outcomes across individuals of various ethnicities, ages, and genders [65]. This study demonstrated that a CCS greater than 100 signifies at least a 7.5% ASCVD risk, regardless of race, age, or gender, suggesting that CCS measurement may be appropriate for patients whose ten-year atherosclerotic vascular disease (ASCVD) risk is <7.5%, or when treatment decisions are uncertain in patients at higher risk because of age only without other conventional risk markers, and whose ASCVD score is discordant with their risk profile. There are a limited number of studies evaluating the risk of CAD in cancer survivors; and the standard ten-year risk assessment score in these patients is difficult to determine due to the presence of unknown risk factors. While cancer and CAD share some common risk factors (age, smoking, obesity, etc.), cancer therapies and radiation further increase that risk. In one study, a CT based approach to detecting CAD in asymptomatic stem-cell transplant (SCT) survivors was used in which they examined CCS with simultaneous CCTA in 20 post SCT recipients [66]. CAD (defined as >50% stenosis) was detected in 4/15 (26.6%) patients that would be considered low risk by conventional FRS classification, highlighting the fact that conventional risk assessments may not be appropriate for this patient population whose cancer puts them at high risk in addition to standard risk factors. In those with CAD, the mean CCS was 55, corresponding to the 75th percentile. Those with no CAD had a mean CCS of 0, corresponding to <1 percentile (*p* < 0.001) [66]. This study concluded that CCS alone (sensitivity of 89% and specificity of 100%) may be adequate for screening and avoids the use of IV contrast. 

HSCT is often preceded by chemotherapy with or without radiation. Mediastinal radiotherapy and anthracycline-containing chemotherapy are major risk factors for the future development of CVD. Two studies to date have examined the relationship between CCS and CAD in asymptomatic patients with Hodgkin Lymphoma (HL) who had survived 15 or more years following treatment with radiation therapy with or without anthracycline-containing chemotherapy [67,68]. In the study by Anderson et al., CCS was higher in the patients with verified CAD compared to those without verified CAD. None of the patients with a CCS of zero had symptomatic CAD [68]. Ten patients out of a total of 47 HL survivors had a CCS score greater than 200, 50% of which underwent revascularization. The study concluded that CCS might be a simple noninvasive screening tool to identify CAD in long-term HL survivors; and that those patients with a score greater than 200 often have CAD, indicating that further investigation with coronary angiography may be justified. While calcified plaques visualized by CCS impart greater stability and are therefore less likely to rupture; it suggests greater possibility of soft unstable plaque elsewhere, more likely to rupture. In addition, higher CCS is associated with higher amounts of coronary stenosis and subsequent coronary events [69,70,71]. Thus, CCS is a great screening tool for risk of future coronary (and indeed cardiovascular) events. Furthermore, coronary calcification is 100% specific for atherosclerotic disease as it has been shown that the presence of CAC excludes the possibility of the artery being normal and free of atherosclerotic disease [72]. More large-scale studies are needed to evaluate the utility of CCT as a screening tool in specific populations prior to HSCT.

### Coronary Computed Tomography Angiogram (CCTA)

CCTA is mainly used for the evaluation of cardiac anatomy, suspected CAD, and for follow up after percutaneous coronary intervention (PCI) or coronary artery bypass grafting (CABG) [73]. As mentioned above, significant atherosclerosis may be present in the absence of calcium deposition. CCTA allows for direct visualization of the coronary arteries and allows for plaque detection, total plaque quantification, and characterization. With CCTA, coronary segments are assessed and classified based on the degree of stenosis severity, and each plaque is classified as calcified, noncalcified, or mixed. Histologic studies indicate that plaque composition has an influence on the pathogenesis and severity of epicardial lesions, regardless of the severity of the underlying stenosis [74]. Most cases of acute coronary syndromes (ACS) are caused by rupture of plaques that did not significantly compromise the area of the coronary lumen prior to the event. Additionally, nonobstructive plaques are more common than severely obstructive plaques [75]. CCTA can identify plaque morphology, an independent risk factor of cardiovascular outcomes. In one study that examined clinical outcomes among patients with nonobstructive CAD, mortality incrementally increased from calcified plaque (1.4%), to partially calcified plaque (3.3%) to noncalcified plaque (9.6%). Thus, the ability of CCTA to identify the presence, severity, and extent of CAD as well as plaque morphology, make it a unique, noninvasive imaging technique in identifying patients suspected of having CAD [76]. In a study that examined coronary angiographic findings in patients initially referred based on prior diagnostic testing, patients that underwent CCTA had a higher proportion of obstructive CAD on coronary angiography compared to stress testing, suggesting utility in more appropriate selection for invasive testing and avoiding unnecessary catheterizations that will show no obstructive CAD [77].

The use of CCTA in HSCT patients has not been well studied. However, in the study of HSCT patients mentioned above that examined CCS with concomitant CCTA, CAD was detected in 4/15 (26.6%) patients that would be considered low risk by conventional FRS classification. There was one patient in this study with a FRS <1% who had a CCS of zero in which nonobstructive plaques were identified on CCTA. This suggests that CCS with CCTA is an acceptable and sensitive screening method amongst asymptomatic low FRS SCT candidates. As mentioned previously, HSCT is often preceded by chemotherapy with or without radiation therapy, which are independent risk factors for cardiovascular disease. One study examined CCS with CCTA in nine patients with HL [78]. Eight of these patients had a CCS above the 75th percentile, and one patient had a score of zero. All eight patients with CCS above the 75th percentile had CAD on CCTA. Three of these patients underwent further investigation, one of which underwent angioplasty, one a two-vessel CABG, and the other a stress echo that was negative. This demonstrates a potential use for screening asymptomatic patients that have previously undergone chemo- and radiation therapy with CCS and CCTA; particularly prior to HSCT.

**Figure 2:**
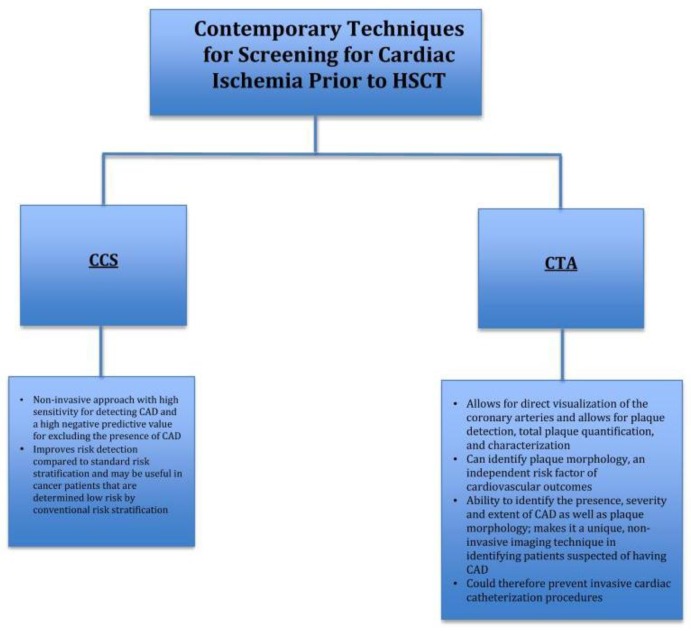
Contemporary techniques for screening for cardiac ischemia prior to HSCT. HSCT, hematopoietic stem cell transplant; CAD, coronary artery disease; CCS, coronary calcium scoring; CCTA, cardiac computed tomography angiography.

Figure 2 provides a summary of contemporary techniques for screening for cardiac ischemia prior to HSCT:

## 7. Conclusions

There are currently no guidelines for comprehensive cardiovascular screening protocols for patients undergoing HSCT. Screening these patients is important because they are at increased risk for developing cardiovascular complications peri-HSCT, possibly leading to increased mortality at a relatively young age. The current method used to screen patients for cardiotoxicity related to chemotherapy is through LVEF assessment by 2D echo; and further testing by stress testing with or without coronary angiography by cardiac catheterization, as warranted. However, cardiac catheterization is somewhat invasive with some associated risks. Emerging technologies such as STE and CMR can potentially detect cardiac dysfunction earlier than conventional LVEF measurements. Furthermore, a noninvasive screening tool, such as CCS with or without CCTA could potentially help to identify HSCT patients at risk for CAD. Future studies are needed that examine major adverse cardiovascular events such as increased mortality that correlate with newer myocardial imaging modalities such as STE and CMR. Similarly, more large-scale prospective studies are needed to determine the prognostic yield of CCS with or without CCTA for CAD assessment prior to HSCT, and confirm their value as screening tests in this population.

## References

[1] Blaes A., Konety S., Hurley P. (2016). Cardiovascular complications of hematopoietic stem cell transplantation. Curr. Treat. Opt. Cardiovasc. Med..

[2] Tonorezos E.J., Stillwell E.E., Calloway J.J., Glew T., Wessler J.D., Rebolledo B.J., Pham A., Steingart R.M., Lazarus H., Gale R.P. (2015). Arrhythmias in the setting of hematopoietic cell transplants. Bone Marrow Transpl..

[3] Sureddi R.K., Amani F., Hebbar P., Williams D.K., Leonardi M., Paydak H., Mheta J.L. (2012). Atrial fibrillation following autologous stem cell transplantation in patients with multiple myeloma: Incidence and risk factors. Ther. Adv. Cardiovasc. Dis..

[4] Murdych T., Weisdorf D. (2001). Serious cardiac complications during bone marrow transplantation at the University of Minnesota, 1977–1997. Bone Marrow Transpl..

[5] Goldberg M.A., Antin J.H., Guinan E.C., Rappeport J.M. (1986). Cyclophosphamide cardiotoxicity: An analysis of dosing as a risk factor. Blood.

[6] Steinherz L.J., Steinherz P.G., Mangiacasale D., O’Reilly R., Allen J., Sorell M., Miller D.R. (1981). Cardiac changes with cyclophosphamide. Med. Pediatr. Oncol..

[7] Armenian S.H., Chow E.J. (2014). Cardiovascular disease in survivors of hematopoietic cell transplantation. Cancer.

[8] Tichelli A., Rovó A., Passweg J., Schwarze C.P., Van Lint M.T., Arat M., Socie G. (2009). Late complications after hematopoietic stem cell transplantation. Expert Rev. Hematol..

[9] Sun C.-L., Francisco L., Kawashima T., Leisenring W., Robison L.L., Baker K.S., Weisdorf D.J., Forman S.J., Bhatia S. (2010). Prevalence and predictors of chronic health conditions after hematopoietic cell transplantation: A report from the bone marrow transplant survivor study. Blood.

[10] Chow E.J., Mueller B.A., Baker K.S., Cushing-Haugen K.L., Flowers M.E.D., Martin P.J., Friedman D.L., Lee S.J. (2011). Cardiovascular hospitalizations and mortality among recipients of hematopoietic stem cell transplantation. Ann. Intern. Med..

[11] Scott J.M., Armenian S., Giralt S., Moslehi J., Wang T., Jones L.W. (2016). Cardiovascular disease following hematopoietic stem cell transplantation: Pathogenesis, detection, and the cardioprotective role of aerobic training. Crit. Rev. Oncol. Hematol..

[12] Scott Baker K., Ness K.K., Steinberger J., Carter A., Francisco L., Burns L.J., Sklar C., Forman S., Weisdorf D., Gurney J.G. (2007). Diabetes, hypertension, and cardiovascular events in survivors of hematopoietic cell transplantation: A report from the bone marrow transplantation survivor study. Blood.

[13] Tichelli A., Bucher C., Rovó A., Stussi G., Stern M., Paulussen M., Halter J., Meyer-Monard S., Heim D., Tsakiris D.A. (2007). Premature cardiovascular disease after allogeneic hematopoietic stem-cell transplantation. Blood.

[14] Cardinale D., Colombo A., Lamantia G., Colombo N., Civelli M., De Giacomi G., Rubino M., Veglia F., Fiorentini C., Cipolla C.M. (2010). Anthracycline-induced cardiomyopathy. J. Am. Coll. Cardiol..

[15] Koene R.J., Prizment A.E., Blaes A., Konety S.H. (2016). Shared risk factors in cardiovascular disease and cancer. Circulation.

[16] Khayata M., Al-Kindi S., Njoroge L., De Lima M.J.G., Oliveira G.H. (2018). Preexisting cardiovascular disease in patients undergoing hematopoietic stem cell transplantation. J. Clin. Oncol..

[17] Tichelli A., Bhatia S., Socié G. (2008). Cardiac and cardiovascular consequences after haematopoietic stem cell transplantation. Br. J. Haematol..

[18] Rhodes M., Lautz T., Kavanaugh-Mchugh A., Manes B., Calder C., Koyama T., Liske M., Parra D., Frangoul H. (2005). Pericardial effusion and cardiac tamponade in pediatric stem cell transplant recipients. Bone Marrow Transpl..

[19] Angelucci E., Mariotti E., Lucarelli G., Baronciani D., Cesaroni P., Durazzi S.M., Galimberti M., Giardini C., Muertto P., Polchi P. (1992). Sudden cardiac tamponade after chemotherapy for marrow transplantation in thalassaemia. Lancet.

[20] Okamoto S. (2017). Current indication for hematopoietic cell transplantation in adults. Hematol. Oncol. Stem Cell Ther..

[21] Qazilbash M.H., Amjad A.I., Qureshi S., Qureshi S.R., Saliba R.M., Khan Z.U., Hosing C., Giralt SA., De Lima M.J., Popat U.R. (2009). Outcome of allogeneic hematopoietic stem cell transplantation in patients with low left ventricular ejection fraction. Biol. Blood Marrow Transpl..

[22] Picard M.H., Popp R.L., Weyman A.E. (2008). Assessment of left ventricular function by echocardiography: A technique in evolution. J. Am. Soc. Echocardiogr..

[23] Foley T.A., Mankad S.V., Anavekar N.S., Bonnichsen C.R., Morris M.F., Miller T.D., Araoz P.A. (2012). Measuring left ventricular ejection fraction—Techniques and potential pitfalls. Eur. Cardiol. Rev..

[24] Lane C., Dorian P., Ghosh N., Radina M., O’Donnell S., Thorpe K., Mangat I., Korley V., Pinter A. (2010). Limitations in the current screening practice of assessing left ventricular ejection fraction for a primary prophylactic implantable defibrillator in southern Ontario. Can. J. Cardiol..

[25] Khouri M.G., Douglas P.S., Mackey J.R., Martin M., Scott J.M., Scherrer-Crosbie M., Jones L.W. (2012). Cancer therapy—Induced cardiac toxicity in early breast cancer. Circulation.

[26] Telli M.L., Hunt S.A., Carlson R.W., Guardino A.E. (2007). Trastuzumab-related cardiotoxicity: Calling into question the concept of reversibility. J. Clin. Oncol..

[27] Adams D., Venkateshvaran A., Alenezi F. (2017). Speckle tracking strain echocardiography: What sonographers need to know!. J. Indian Acad. Echocardiogr. Cardiovasc. Imaging.

[28] Levy P.T., Machefsky A., Sanchez A.A., Patel M.D., Rogal S., Fowler S., Yaeger L., Hardi A., Holland M.R., Hamvas A. (2016). Reference ranges of left ventricular strain measures by two-dimensional speckle-tracking echocardiography in children: A systematic review and meta-analysis. J. Am. Soc. Echocardiogr..

[29] Carlos Plana J., Galderisi M., Barac A., Ewer M.S., Ky B., Scherrer-Crosbie M., Ganame J., Sebag I.A., Agler D.A., Badano L.P. Expert Consensus for Multimodality Imaging Evaluation of Adult Patients during and after Cancer Therapy: A Report from the American Society of Echocardiography and the European Association of Cardiovascular Imaging from the Cleveland Clinic 2014.

[30] Baratta S., Damiano M.A., Marchese M.L., Trucco J.I., Rizzo M.M., Bernok F., Cheitman D., Olano D., Rojas M., Hita A. (2013). Serum markers, conventional doppler echocardiography and two-dimensional systolic strain in the diagnosis of chemotherapy-induced myocardial toxicity. Argent. J. Cardiol..

[31] Thavendiranathan P., Poulin F., Lim K.-D., Plana J.C., Woo A., Marwick T.H. (2014). Use of myocardial strain imaging by echocardiography for the early detection of cardiotoxicity in patients during and after cancer chemotherapy: A systematic review. J. Am. Coll. Cardiol..

[32] Sawaya H., Sebag I.A., Plana J.C., Januzzi J.L., Ky B., Cohen V., Gosavi S., Carver J.R., Wiegers S.E., Martin R.P. (2011). Early detection and prediction of cardiotoxicity in chemotherapy-treated patients. Am. J. Cardiol..

[33] Sengeløv M., Jørgensen P.G., Skov Jensen J., Bruun N.E., Olsen F.J., Fritz-Hansen T., Nochioka K., Biering-Sorensen T. Global Longitudinal Strain Is a Superior Predictor of All-Cause Mortality in Heart Failure with Reduced Ejection Fraction 2015. http://imaging.onlinejacc.org/content/jimg/8/12/1351.full.pdf.

[34] Farsalinos K.E., Daraban A.M., Ünlü S., Thomas J.D., Badano L.P., Voigt J.-U. (2015). Head-to-head comparison of global longitudinal strain measurements among nine different vendors. J. Am. Soc. Echocardiogr..

[35] Yoon J.-H., Kim H.J., Lee E.-J., Moon S., Lee J.Y., Lee J.W., Chung N.G., Cho B., Kim H.K. (2015). Early left ventricular dysfunction in children after hematopoietic stem cell transplantation for acute leukemia: a case control study using speckle tracking echocardiography. Korean Circ. J..

[36] Risum N., Ali S., Olsen N.T., Jons C., Khouri M.G., Lauridsen T.K., Samad Z., Velazquez E.J., Sogaard P., Kisslo J. (2012). Variability of global left ventricular deformation analysis using vendor dependent and independent two-dimensional speckle-tracking software in adults. J. Am. Soc. Echocardiogr..

[37] Sandner T.A., Houck P., Runge V.M., Sincleair S., Huber A.M., Theisen D., Reiser M.F., Wintersperger B.J. (2008). Accuracy of accelerated cine MR imaging at 3 Tesla in longitudinal follow-up of cardiac function. Eur. Radiol..

[38] Grothues F., Smith G.C., Moon J.C., Bellenger N.G., Collins P., Klein H.U., Pennell D.J. (2002). Comparison of interstudy reproducibility of cardiovascular magnetic resonance with two-dimensional echocardiography in normal subjects and in patients with heart failure or left ventricular hypertrophy. Am. J. Cardiol..

[39] Wassmuth R., Lentzsch S., Erdbruegger U., Schulz-Menger J., Doerken B., Dietz R., Friedrich M.G. (2001). Subclinical cardiotoxic effects of anthracyclines as assessed by magnetic resonance imaging—A pilot study. Am. Heart J..

[40] Ylänen K., Poutanen T., Savikurki-Heikkilä P., Rinta-Kiikka I., Eerola A., Vettenranta K. (2013). Cardiac magnetic resonance imaging in the evaluation of the late effects of anthracyclines among long-term survivors of childhood cancer. J. Am. Coll. Cardiol..

[41] Sutton-Tyrrell K., Najjar S.S., Boudreau R.M., Venkitachalam L., Kupelian V., Simonsick E.M., Havlik R., Lakatta E.G., Spurgeon H., Kritchevsky S. (2005). Elevated aortic pulse wave velocity, a marker of arterial stiffness, predicts cardiovascular events in well-functioning older adults. Circulation.

[42] Chaosuwannakit N., D’Agostino R., Hamilton C.A., Lane K.S., Ntim W.O., Lawrence J., Melin S.A., Ellis L.R., Torti F.M., Little W.C. (2010). Aortic stiffness increases upon receipt of anthracycline chemotherapy. J. Clin. Oncol..

[43] Drafts B.C., Twomley K.M., D’Agostino R., Lawrence J., Avis N., Ellis L.R., Thohan V., Jordan J., Melin S.A., Torti F.M. (2013). Low to moderate dose anthracycline-based chemotherapy is associated with early noninvasive imaging evidence of subclinical cardiovascular disease. JACC Cardiovasc. Imaging.

[44] Schelbert E.B., Fonarow G.C., Bonow R.O., Butler J., Gheorghiade M. (2014). Therapeutic targets in heart failure. J. Am. Coll. Cardiol..

[45] Swynghedauw B. (1999). Molecular mechanisms of myocardial remodeling. Physiol. Rev..

[46] Jordan J.H., Vasu S., Morgan T.M., D’Agostino R.B., Meléndez G.C., Hamilton C.A., Arai A.E., Liu S., Liu C.Y., Lima J.A. (2016). Anthracycline-associated T1 mapping characteristics are elevated independent of the presence of cardiovascular comorbidities in cancer survivors. Circ. Cardiovasc. Imaging.

[47] Dekker D.L., Piziali R.L., Dong E. (1974). A system for ultrasonically imaging the human heart in three dimensions. Comput. Biomed. Res..

[48] Gopal A.S., Keller A.M., Rigling R., King D.L., King D.L. (1993). Left ventricular volume and endocardial surface area by three-dimensional echocardiography: Comparison with two-dimensional echocardiography and nuclear magnetic resonance imaging in normal subjects. J. Am. Coll. Cardiol..

[49] Wang X.-F., Deng Y.-B., Nanda N.C., Deng J., Miller A.P., Xie M.-X. (2003). Live three-dimensional echocardiography: imaging principles and clinical application. Echocardiography.

[50] Jenkins C., Bricknell K., Chan J., Hanekom L., Marwick T.H. (2007). Comparison of two- and three-dimensional echocardiography with sequential magnetic resonance imaging for evaluating left ventricular volume and ejection fraction over time in patients with healed myocardial infarction. Am. J. Cardiol..

[51] Caiani E.G., Corsi C., Sugeng L., MacEneaney P., Weinert L., Mor-Avi V., Lang R.M. (2006). Improved quantification of left ventricular mass based on endocardial and epicardial surface detection with real time three dimensional echocardiography. Heart.

[52] Hare J.L., Jenkins C., Nakatani S., Ogawa A., Yu C.-M., Marwick T.H. (2007). Feasibility and clinical decision-making with 3D echocardiography in routine practice. Heart.

[53] Thavendiranathan P., Grant A.D., Negishi T., Plana J.C., Popović Z.B., Marwick T.H. (2013). Reproducibility of echocardiographic techniques for sequential assessment of left ventricular ejection fraction and volumes. J. Am. Coll. Cardiol..

[54] Mahabadi A.A., Rischpler C. (2018). Cardiovascular imaging in cardio-oncology. J. Thorac. Dis..

[55] Sicari R., Nihoyannopoulos P., Evangelista A., Kasprzak J., Lancellotti P., Poldermans D., Voigt J.U., Zamorano J.L. (2008). Stress echocardiography expert consensus statement: European Association of Echocardiography (EAE) (a registered branch of the ESC). Eur. J. Echocardiogr..

[56] Lin G.S., Hines H.H., Grant G., Taylor K., Ryals C. (2006). Automated quantification of myocardial ischemia and wall motion defects by use of cardiac SPECT polar mapping and 4-dimensional surface rendering. J. Nucl. Med. Technol..

[57] Gayed I.W., Liu H.H., Yusuf S.W., Komaki R., Wei X., Wang X., Chang J.Y., Swafford J., Broemeling L., Liao Z. (2006). The prevalence of myocardial ischemia after concurrent chemoradiation therapy as detected by gated myocardial perfusion imaging in patients with esophageal cancer. J. Nucl. Med..

[58] Imbert L., Marie P.-Y. (2016). CZT cameras: A technological jump for myocardial perfusion SPECT. J. Nucl. Cardiol..

[59] Hsiao E.M., Rybicki F.J., Steigner M. (2010). CT coronary angiography: 256-slice and 320-detector row scanners. Curr. Cardiol. Rep..

[60] Greenland P., Kizilbash M.A. (2005). Coronary computed tomography in coronary risk assessment. J. Cardiopulm. Rehabil..

[61] Detrano R., Guerci A.D., Carr J.J., Bild D.E., Burke G., Folsom A.R., Liu K., Shea S., Szklo M., Bluemke D.A. (2008). Coronary calcium as a predictor of coronary events in four racial or ethnic groups. N. Engl. J. Med..

[62] Elias-Smale S.E., Proença R.V., Koller M.T., Kavousi M., van Rooij F.J.A., Hunink M.G., Steyerberg E.W., Hofman A., Oudkerk M., Witteman J.C. (2010). Coronary Calcium score improves classification of coronary heart disease risk in the elderly. J. Am. Coll. Cardiol..

[63] Erbel R., Möhlenkamp S., Moebus S., Schmermund A., Lehmann N., Stang A., Dragano N., Grönemeyer D., Seibel R., Kälsch H. (2010). Coronary risk stratification, discrimination, and reclassification improvement based on quantification of subclinical coronary atherosclerosis. J. Am. Coll. Cardiol..

[64] Stone N.J., Robinson J.G., Lichtenstein A.H., Bairey Merz C.N., Blum C.B., Eckel R.H., Goldberg A.C., Gordon D., Levy D., Lloyd-Jones D.M. (2014). 2013 ACC/AHA guideline on the treatment of blood cholesterol to reduce atherosclerotic cardiovascular risk in adults. Circulation.

[65] Budoff M.J., Young R., Burke G., Jeffrey Carr J., Detrano R.C., Folsom A.R., Kronmal R., Lima J.A.C., Liu K.J., McClelland R.L. (2018). Ten-year association of coronary artery calcium with atherosclerotic cardiovascular disease (ASCVD) events: The multi-ethnic study of atherosclerosis (MESA). Eur. Heart J..

[66] Jain N.A., Chen M.Y., Shanbhag S., Lu K., Pophali P.A., Ito S., Koklanaris E., Hourigan C.S., Barrett J.A., Battiwalla M. (2014). Contrast enhanced cardiac CT reveals coronary artery disease in 45% of asymptomatic allo-SCT long-term survivors. Bone Marrow Transpl..

[67] Agatston A.S., Janowitz W.R., Hildner F.J., Zusmer N.R., Viamonte M., Detrano R. (1990). Quantification of coronary artery calcium using ultrafast computed tomography. J. Am. Coll. Cardiol..

[68] Andersen R., Wethal T., Günther A., Fosså A., Edvardsen T., Fosså S.D., Kjekshus J. (2010). Relation of coronary artery calcium score to premature coronary artery disease in survivors> 15 years of Hodgkin’s lymphoma. Am. J. Cardiol..

[69] Iwasaki K., Matsumoto T. (2016). Relationship between coronary calcium score and high-risk plaque/significant stenosis. World J. Cardiol..

[70] Budoff M.J., Nasir K., McClelland R.L., Detrano R., Wong N., Blumenthal R.S., Kondos G., Kronmal R.A. (2009). Coronary calcium predicts events better with absolute calcium scores than age-sex-race/ethnicity percentiles: MESA (Multi-Ethnic Study of Atherosclerosis). J. Am. Coll. Cardiol..

[71] Yeboah J., Delaney J.A., Nance R., McClelland R.L., Polak J.F., Sibley C.T., Bertoni A., Burke G.L., Carr J.J., Herrington D.M. (2014). Mediation of cardiovascular risk factor effects through subclinical vascular disease. Arterioscler. Thromb. Vasc. Biol..

[72] Rumberger J.A., Simons D.B., Fitzpatrick L.A., Sheedy P.F., Schwartz R.S. (1995). Coronary artery calcium area by electron-beam computed tomography and coronary atherosclerotic plaque area. Circulation.

[73] Budoff M.J., Achenbach S., Blumenthal R.S., Carr J.J., Goldin J.G., Greenland P., Guerci A.D., Lima J.A., Rader D.J., Rubin G.D. (2006). Assessment of coronary artery disease by cardiac computed tomography. Circulation.

[74] Carità P., Guaricci A.I., Muscogiuri G., Carrabba N., Pontone G. (2018). Prognostic value and therapeutic perspectives of coronary CT angiography: A literature review. BioMed Res. Int..

[75] Falk E., Shah P.K., Fuster V. (1995). Coronary plaque disruption. Circulation.

[76] Ahmadi N., Nabavi V., Hajsadeghi F., Flores F., French W.J., Mao S.S., Shavelle D., Ebrahimi R., Budoff M. (2011). Mortality incidence of patients with non-obstructive coronary artery disease diagnosed by computed tomography angiography. Am. J. Cardiol..

[77] Patel M.R., Dai D., Hernandez A.F., Douglas P.S., Messenger J., Garratt K.N., Maddox T.M., Peterson E.D., Roe M.T. (2014). Prevalence and predictors of nonobstructive coronary artery disease identified with coronary angiography in contemporary clinical practice. Am. Heart J..

[78] Rademaker J., Schöder H., Ariaratnam N.S., Strauss H.W., Yahalom J., Steingart R., Oeffinger K.C. (2008). Coronary artery disease after radiation therapy for Hodgkin’s lymphoma: Coronary CT angiography findings and calcium scores in nine asymptomatic patients. Am. J. Roentgenol..

